# A Molecular Study of Aspirin and Tenofovir Using Gold/Dextran Nanocomposites and Surface-Enhanced Raman Spectroscopy

**DOI:** 10.3390/molecules27082554

**Published:** 2022-04-15

**Authors:** Setumo Lebogang Thobakgale, Saturnin Ombinda-Lemboumba, Patience Mthunzi-Kufa

**Affiliations:** 1 National Laser Centre, Council for Scientific and Industrial Research, P.O. Box 395, Pretoria 0001, South Africa; sombindalemboumba@csir.co.za (S.O.-L.); pmthunzikufa@csir.co.za (P.M.-K.); 2 School of Chemistry and Physics, College of Agriculture, Engineering and Science, University of Kwa-Zulu Natal, University Road, Westville, Durban 3630, South Africa

**Keywords:** aspirin, tenofovir, gold nanoparticles, dextran, chemical sensors, SERS

## Abstract

In this study, we show how surface enhanced Raman spectroscopy (SERS) can be used to monitor the molecular behaviour of aspirin and tenofovir as a means of screening medication for quality control purposes. Gold-coated slides combined with gold/dextran nanoaggregates were used to provide signal enhancement of the drugs using SERS. Aspirin (10% *w*/*v*) and tenofovir (20% *v*/*v*) were analysed in the presence of the nanomaterials to determine trends in molecular response to changes in gold/dextran concentrations. Qualitative analysis of the functional groups showed specific trends where the peak area increased with polarizability, electron density and decreased atomic radii. Steric hinderance effects also affected the trends in peak area due to the amount of gold/dextran nanoparticles in solution. Statistical analysis provided accurate and precise linear relationships (R^2^ = 0.99) for the ester and adenine functional groups of aspirin and tenofovir, respectively. From the above findings, the combined use of gold nano-scaffolds and gold/dextran nanomaterials amplified the Raman signal from the drugs to allow for systematic evaluation of their molecular properties. Although more experiments to correlate the findings are still needed, this SERS approach shows great potential as a screening method in the quality control of medications.

## 1. Introduction

Raman spectroscopy is a photonics technique that uses light to investigate the molecular properties of an analyte. When monochromatic light interacts with matter, a scattering event occurs which is used to identify the molecular bond that is responding to the photons from the light. Although this method is non-destructive and non-invasive, its inherent limitation is that it produces a low signal, which has been approximated to be 0.01% of the laser intensity [1,2]. Many efforts have been explored to overcome this limitation and to produce higher Raman signals, one such approach being surface enhanced Raman spectroscopy (SERS) [3]. This method incorporates nanomaterials as support structures that amplify the Raman signal [10^4^–10^8^] to achieve improved sensitivity, selectivity and detection limit [4]. Although the exact mechanism of SERS is still under discussion, it is widely accepted that the surface enhancement effect arises from two processes: electromagnetic and chemical enhancement [5,6]. In the case of electromagnetic enhancement (EM), the SERS effect occurs from the interaction between the incident laser photons and the surface plasmons, which are the collective oscillating frequencies of the conducting electrons found on the surface of the metallic nanostructure [7,8]. The efficiency of this method relies on the distance between the analyte and the metal nanoparticle (NP); thus, only molecules which are near the NP, referred to as the “hotspot”, will experience the maximum Raman signal amplification [9,10]. The chemical enhancement model (CM) employs molecular interactions such as covalent bonding, charge-selective mechanism, hydrophobic interactions and π-π stacking between the analyte and the NP to increase the Raman cross-scattering area, thereby improving the Raman signal intensity [11,12]. 

After many years of disputes, it is generally accepted that SERS effects primarily originate from the coupling of the incident laser light with the localized surface plasmon resonance (LSPR) of the nanostructured metal surface of nanoparticles [13]. This results in a gigantic signal enhancement which enhances the Raman spectra of the analytes under investigation. Surface plasmon resonance happens as a result of the collective oscillations of valence electrons in resonance with the incident light. This is localized by the dimensions of metallic nanoparticles with the dimensions smaller than the wavelength, therefore creating a locally amplified electromagnetic (EM) field which can be further enhanced in regions between nanoparticles because of near-field coupling [14].

Gold nanoparticles (AuNPs) have been used extensively as sensing platforms because their atomic structure is suited for inducing the SERS effect through the EM and CM models [15,16]. The bench-top synthesis of AuNPs for sensing applications has been largely explored using the self-assembly method (SAM), where the positively charged AuNPs are allowed to assemble onto negatively charged surfaces such as glass and various polymers [17,18,19]. Chemical fabrication with AuNPs also provides a surface roughness which is critical in facilitating physical adsorption of the analyte onto the substrate [20,21]. Another SERS approach involves preparation of aggregated nanoparticles in a solution and then combining with the analyte to increase the chances of analyte/nanoparticle interaction to assist the nanomaterial-coated scaffolds with signal enhancement. In this fashion, intermolecular forces such as hydrogen bonding, dispersion, van der Waals, ion-dipole and dipole–dipole can bring the analyte closer to the hotspot for signal enhancement [22]. Atomic properties such as polarizability, electron density, electronegativity and atomic radii play a major role in how the molecules respond to a change in EM field caused by the Raman laser [23,24,25]. Furthermore, the molecular structures of the analyte and gold/polymer nanoparticle solutions largely dictate the efficiency of their interaction and the subsequent signal amplification [26,27,28]. 

SERS has become useful in the pharmaceutical space where chemical sensors and aggregated solutions have been synthesized for the purpose of determining molecular fingerprints with improved sensitivity. For example, star-like gold nanoparticles functionalized with L-cysteine and supported with graphene oxide were used for therapeutic drug monitoring of Paclitaxel, a drug used to treat a variety of cancer agents. Silver nanomaterial colloids were used to detect Benzylpenicillin (bacterial treatment), while immunoglobin G (blood antibody) was monitored using nanoscale graphene oxide and gold nanoparticles [29,30,31]. In this study, we evaluate the molecular behaviour of aspirin (acetylsalicylic acid, ASA) a well-known pain killer and tenofovir (tenofovir disoproxil fumarate), an antiretroviral medication widely used to treat HIV in third-world countries. Standard solutions of the drugs were analysed using SERS, to determine if the combination of Au/dextran nanoparticles and Au-coated glass slides are a suitable method for screening of medication supplied to low resource-based countries. 

## 2. Results

### 2.1. SEM of Gold-Coated Glass Slides Using Self-Assembly Method

**Figure 1:**
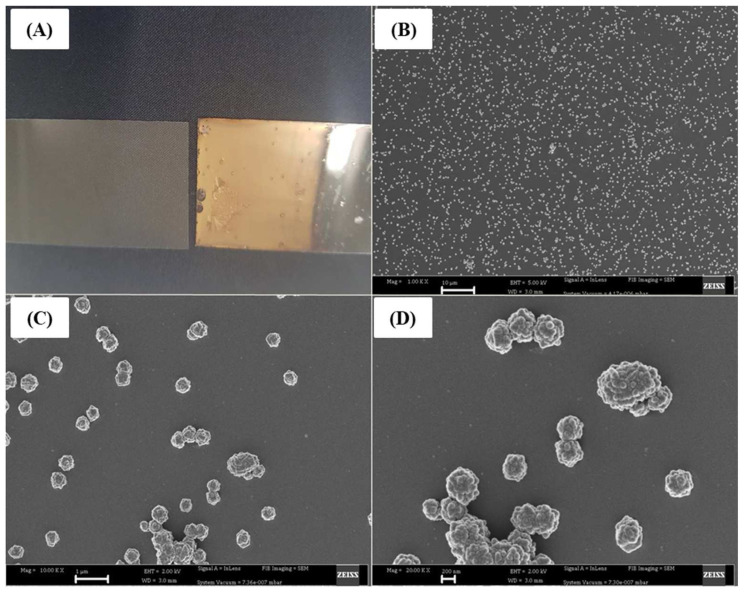
Images of a gold-coated slide after self-assembly. (**A**) Visual image of plain and coated slides. (**B**–**D**) SEM images of gold nanoparticles at 1, 10 and 20× magnification, respectively, acquired from a Zeiss scanning electron microscope.

Following synthesis of the gold-coated slides, scanning electron microscopy was performed to evaluate the morphology. Figure 1 below shows the images obtained after the self-assembly of gold nanoparticles on glass slides.

In Figure 1A, the difference between plain glass (left) and gold-coated glass (right) is depicted where a reddish/brown layer is visible on the coated sample, indicating the presence of AuNPs. Figure 1B is a 1× magnification of AuNPs covering most of the imaged area. At 10× magnification (1C), the spherically shaped nanoparticles are dispersed randomly across the image. Lastly, Figure 1D is a 20× image which shows the approximate size of the particles to be 200–250 nm. Although the SEM images show that there was good deposition of the gold nanoparticles, the nanoparticles lack uniformity, which can be attributed to the hydrophilic glass used for coating. More hydrophilic surfaces have been shown to produce better uniform structures [16].

### 2.2. UV-Vis Spectroscopy of Au/Dextran Nanocomposites

**Figure 2:**
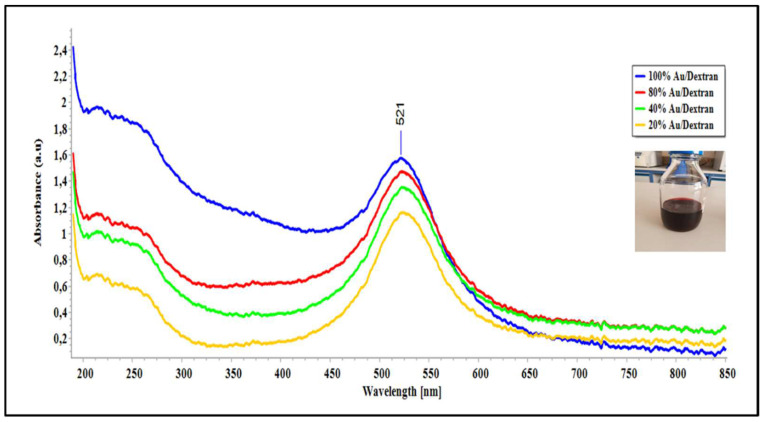
UV-Vis spectra of gold/dextran nanoparticles at 100%, 80%, 40% and 20% concentration. Spectra were obtained using an insert: actual image of the nanoparticles.

Gold nanoparticles coated with dextran DEAE prepared by chemical reduction were analysed with UV-Vis spectroscopy to confirm their presence through measuring changes in absorption. Figure 2 below shows the spectra obtained from Au/dextran nanoparticles at various concentrations.

The spectral image in Figure 2 shows the absorption profiles of gold/dextran nanoparticles diluted from a stock solution prepared as described in the methods section. All the samples show an absorption peak at 521 nm wavelength, which is characteristic for gold nanoparticles. Second, the absorbance increases with concentration of the samples as expected from the Beer-Lambert law [32]. The insert depicts a wine-red solution collected after synthesis, which is known for gold nanoparticles. Lastly, the spectra show minimum red shifting, which implies a narrow distribution in nanoparticle size. Actual nanoparticle sizes could not be determined at the time of the experiment and were thus not considered as a parameter during analysis. 

### 2.3. Surface Enhanced Raman Spectroscopy on Acetylsalicylic Acid (ASA)

**Figure 3:**
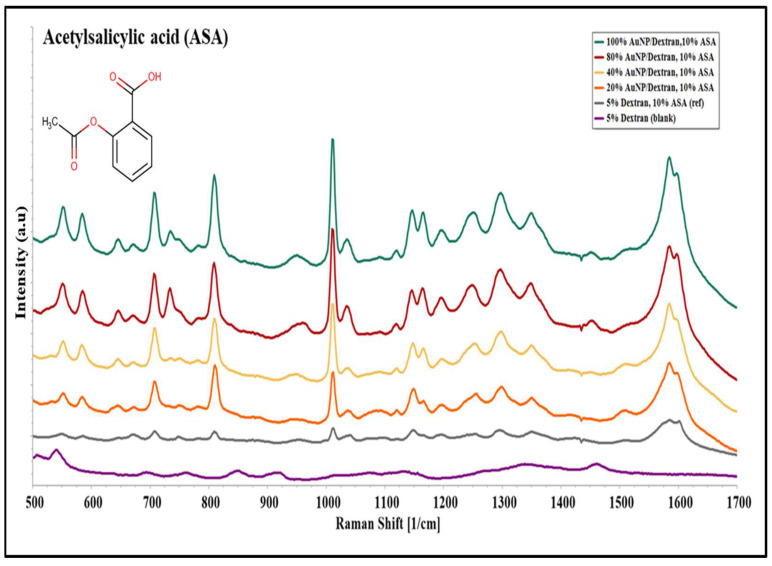
SERS spectra of 10% acetylsalicylic acid (ASA) prepared in Au/dextran NP solutions of 20–100% concentrations; 10% ASA in 5% Dextran was used as a reference, and 5% Dextran solution was used as a blank. Spectra were acquired on a BW Tek Raman microscope.

ASA solutions prepared in various concentrations of Au/dextran NP (Section 2.3) were analysed using SERS to confirm the expected ASA functional groups. Figure 3 below shows a group of Raman spectra acquired at various nanoparticle concentrations on top of a gold-coated microscope slide.

Raman spectra observed in Figure 3 were compared with published literature values to determine the source of the peaks that are observed. For blank samples, no peaks of significant intensity relating to ASA were observed. Reference samples containing 10% ASA in 5% dextran produced fewer peaks of low intensity compared to nanoparticle samples. In the case of ASA in Au/dextran solution, strong peaks such as the rocking mode of the carboxylic group were detected at a Raman shift of 551 cm^−1^, along with the bending mode of carbon and hydrogen bonds (CH) in the 705–837 cm^−1^ spectral region. The ester group emerged as a stretching mode at a shift of 1016 cm^−1^, adjacent to the weak ring breathing mode of the phenyl group found at 1036 cm^−1^. Further down the spectra, the bending mode of CH reappeared at 1154 and 1191 cm^−1^ followed by the carboxylic stretching mode detected at 1293 cm^−1^. Lastly, the stretching mode of the carbons of the phenyl group was found at 1585 cm^−1^. By comparison, only the 809, 1011, 1146 and 1585 cm^−1^ Raman peaks were observed in the reference samples, while the contribution from dextran after background subtraction was seen only in the carbon/hydrogen bonds [33,34]. Table 1 below summarizes the Raman peaks obtained in the ASA experiments.

From the above results, specific peaks relating to functional groups of aspirin were processed further for qualitative and statistical analysis.

### 2.4. Surface Enhanced Raman Spectroscopy on Tenofovir Disoproxil Fumarate

**Figure 4:**
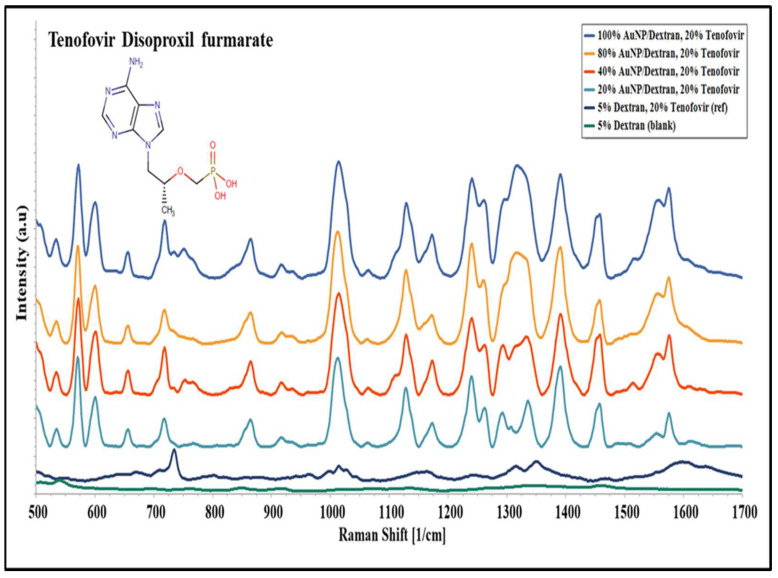
SERS spectra of 20% tenofovir disoproxil fumarate (tenofovir) prepared in Au/dextran NP solutions of 20–100% concentrations; 20% tenofovir in 5% dextran was used as a reference, and 5% dextran solution was used as a blank. Spectra were acquired on BW Tek Raman microscope.

Tenofovir disoproxil fumarate (tenofovir) solutions prepared in various concentrations of Au/dextran NP were analysed using SERS to confirm their characteristic functional groups. Figure 4 below shows a group of Raman spectra acquired at various nanoparticle concentrations on top of a gold-coated microscope slide.

The spectra observed in Figure 4 were compared with the literature to explain the peaks obtained. In the 500–600 cm^−1^ region, the carbon double bonds from the adenine ring were found followed by the Raman peak from the whole adenine ring at 725 cm^−1^. In addition, the tenofovir skeletal molecule is observed at 864 cm^−1^ vibrating in-plane stretching, while a strong peak from the stretching vibration mode caused by the phosphate group appears at 1014 cm^−1^. The 1110–1190 cm^−1^ region constitutes carbon hydrogen (CH) bonds in various modes followed by a combination of carbon nitrogen bonds (CN) and the amine group (CNH_2_) stretching modes appearing in the 1260–1390 cm^−1^ region. Furthermore, a bending mode arising from the primary amine (NCH) and methyl group was found in the 1456 cm^−1^ region. Lastly, CNH bending and scissoring from the amino group appeared in the 1500–1600 cm^−1^ region towards the end of the spectrum. Table 2 below summarizes the Raman spectra obtained from the tenofovir experiments [35,36].

From the above results, Raman peaks unique to functional groups of tenofovir were evaluated further for qualitative and statistical analyses. 

### 2.5. Qualitative and Statistical Analysis of SERS Peaks on ASA

**Figure 5:**
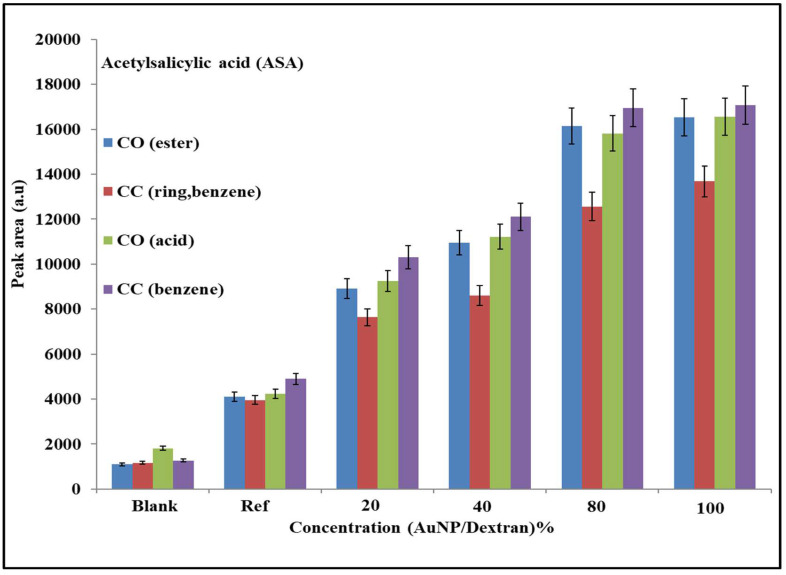
Histogram of ASA functional groups. The plot shows a peak area (PA) vs. Au/dextran NP concentration. The blank sample is 5% dextran, while the reference is 10% ASA in 5% Au/dextran NP solution; 5% error bars are shown for each analysis.

In this section, a qualitative analysis on both drugs was performed to evaluate the changes in molecular behaviour as a result of interactions with the Au/dextran nanoparticles in the presence of AuNP-coated scaffolds. SERS peaks were chosen based on the functional groups unique to each drug, with no chance of peak overlap arising from the dextran functional groups. Figure 5 is a histogram of ASA functional groups under different Au/dextran concentrations. Functional groups such as the CO from the ester, the benzene group and CO from the carboxylic acid were compared based on the molecular characteristics discussed in Section 1.

**Figure 6:**
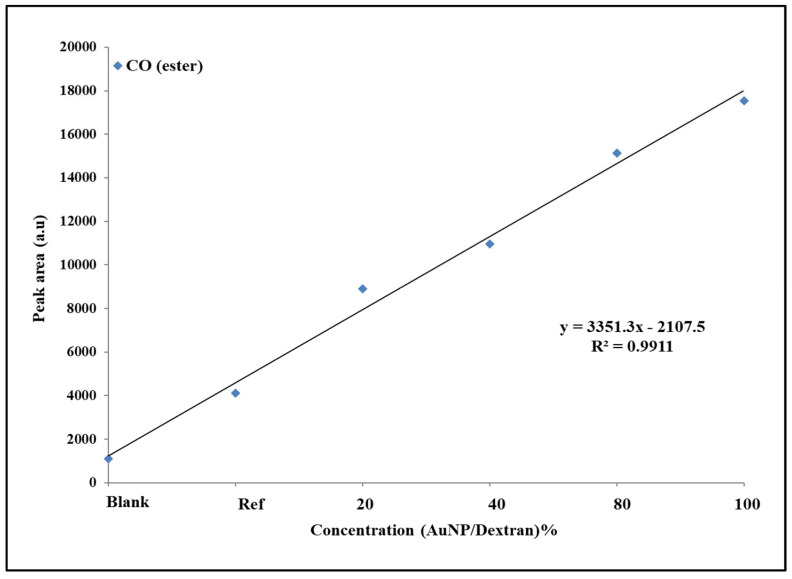
Linear plot of PA (ester) vs. Au/dextran concentration. The blank sample is 5% dextran, while the reference is 10% ASA in 5% Au/dextran NP solution. Experiments were performed in triplicate for each group. (*n* = 3, SD 1.5–2.5). R^2^ = 0.99.

The graph in Figure 5 compares the changes in peak area of the ASA functional groups as a result of the nanomaterial-assisted signal enhancement effects. As seen from left to right, the overall trend of peak areas of all the functional groups increases with Au/dextran NP concentration. Within a group, the benzene peak area (red) is the lowest, while the CC from the benzene (purple) shows the highest peak area values. The oxygen-containing ester and carboxylic groups show similar PA trends, although the latter is consistently higher across all the sample groups. Statistical analysis of the ester and benzene groups was performed to evaluate the relationship between peak area and Au/dextran concentration. Figure 6 below is a peak area vs. concentration plot of the ester group belonging to ASA.

The average peak area values for each ester group were plotted against concentrations of the Au/dextran solutions. A linear relationship between the two parameters was observed with a R^2^ value of 0.99. A The standard deviations (SD) and relative standard deviations (RSD) of each sample group is provided in the supplementary documents.

### 2.6. Qualitative and Statistical Analysis of SERS Peaks on Tenofovir

**Figure 7:**
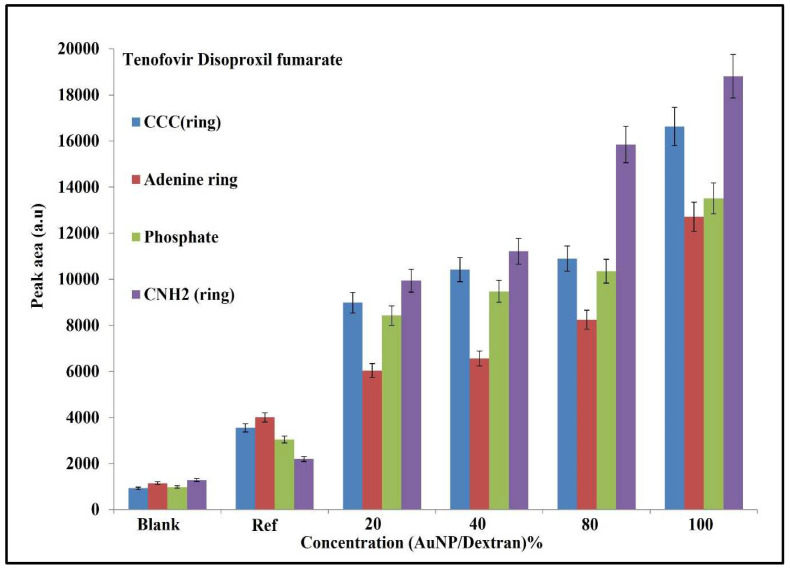
Histogram of tenofovir functional groups. The plot shows a peak area (PA) vs. AuNP/dextran concentration. The blank sample is 5% dextran, while the reference is 20% tenofovir in 5% AuNP/dextran solution; 5% error bars are shown for each analysis.

A similar evaluation was carried out on Raman spectra obtained from the tenofovir experiments. Figure 7 below is a histogram of the functional groups associated with tenofovir. Such groups include the CCC bond of the adenine ring, the entire adenine ring, the phosphate group and primary amine (CNH_2_). 

**Figure 8:**
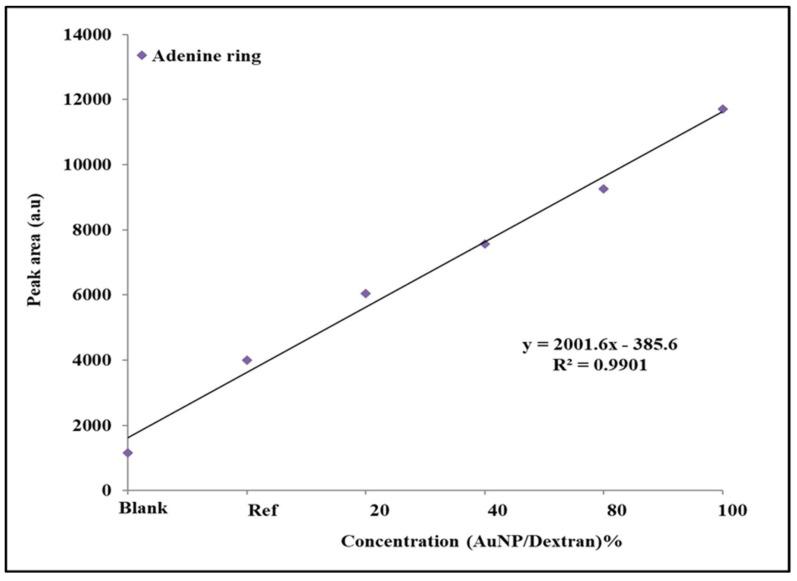
Linear plot of PA (ester) vs. Au/dextran concentration. The blank sample is 5% dextran, while the reference is 20% tenofovir in 5% Au/dextran NP solution. Experiments were performed in triplicate for each group. (*n* = 3, SD 1.5–2.5). R^2^ = 0.99.

The peak area trend shows an increase from left to right, with the blank showing low signals associated with the above-mentioned functional groups. In the blank sample, the phosphate peak area shows the lowest value, while the primary amine has the highest. The reference group has a different trend, with the amine showing the lowest PA value, while the adenine group has the highest. For samples containing Au/dextran nanoparticles, the adenine group shows the lowest PA value across all the concentrations, while the primary amine has the highest. The CCC group of the adenine ring displayed the second highest peak area value, with a small difference observed between 40% and 80% samples. The same trend is observed in the phosphate functional group. Statistical analysis of the adenine ring was performed to evaluate the relationship between peak area and Au/dextran concentration (Figure 8).

The average peak area values for each adenine group concentration were plotted against concentration of the Au/dextran solutions. A linear relationship between the two parameters was observed with a R^2^ value of 0.99. The standard deviations (SD) and relative standard deviations (RSD) of each sample group are provided in the Supplementary Materials.

## 3. Discussion

### 3.1. Molecular Evaluation of Acetylsalicylic Acid

The results from the ASA experiments were evaluated by focusing on the intermolecular forces that exist between molecules that are in proximity. The interactions between the ASA molecules and the Au/dextran nanoparticle solutions were observed through the significant signal enhancement of the Raman spectra compared to the reference samples. From a molecular perspective, this can be explained by considering the highly polarized functional groups of the dextran polymer, which engage in hydrogen bonding between the lone pairs of the epoxy oxygens and the hydrogens of the ASA [37]. Hydrogen bonding also occurs between the oxygen atoms of ASA with the hydrogens of the dextran polymer. This interaction keeps the drug near the gold nanoparticles where the laser-induced plasmon effects from both the solution and the scaffold cause signal enhancement. The histograms in Section 2.5 showed that the peak area trends for the reference and the Au/dextran samples increase in the order of benzene (CC) > CO (acid) > CO (ester) > benzene (ring), which is explained by a few factors. A benzene ring bares the highest electron density, making it the most polarizable moiety within the group. Furthermore, the carbons bonded to the ester and the carboxylic groups in the ortho position experience a dipole moment unlike those in the rest of the ring, making them more receptive to bond stretching, which caused the highest peak area response at 1557 cm^−1^ [33]. Molecular orientation is also a major contributor, as it mostly favours a positioning in space with the least steric hinderance, thus influencing which functional groups receive access to the SERS hotspot [38]. For example, the carboxylic acid and ester appeared to show similar values in peak area within the same concentration group. However, the acid produced higher PA values overall, probably as a result of the difference in molecular size between the ester methyl group and the smaller hydrogen of the acid where the latter was allowed more access to the SERS hotspot to produce an amplified signal. The benzene ring breathing mode was the lowest in the series because it was mostly engaged in the stretching mode explained above while experiencing relatively higher steric hinderance as well. Statistical analysis of the ASA ester response to an increase in Au/dextran content was linear in shape, with a high R^2^ and low SD and RSD per concentration group. These findings prove that the combination of the gold scaffold and the Au/dextran nanoparticle solution was able to detect and amplify the ASA signal with good accuracy and precision.

### 3.2. Molecular Evaluation of Tenofovir

The tenofovir results were studied in a similar method to explain the trends in peak area that were observed. From the histogram data (Figure 7), the reference shows a high adenine ring response and a low CNH_2_ in terms of PA values. In this case, the presence of the gold nanoparticles on the scaffold are assumed to apply ion–dipole forces to the vast number of lone pairs that are available on the adenine with minimum steric hinderance; hence, it was also the second highest with the CCC bond. The amine group was lowest in the reference because polarizability of the phosphate is more probable due to the large atomic radii of phosphorus compared to nitrogen. When the Au/dextran solution was introduced, the peak area increased in the order of amine (CNH_2_) > CCC (adenine) > phosphate > adenine ring. The change in trend can be attributed to the presence of Au/dextran nanoparticles. In this environment, the lone pairs of the amine group interact with the hotspots more frequently through hydrogen bonding with the dextran hydrogens, while the amine hydrogens pair with the dextran oxygen atoms. Furthermore, the conditions that effect steric behaviour are changed in the presence of Au/dextran, leading to a change in preferred functional groups. As such, the less bulky amine group received more exposure to the hotspot followed by the carbons of the adenine molecule which are near the amine group. The phosphate group was third highest in the trend, likely due to the steric hinderance which affects atoms of higher atomic radii and electron density. The same rationale explains the position of the adenine ring PA, which is bulky and contains a large electron density, which places it lowest in the trend when Au/dextran nanoparticles are present. The adenine ring was evaluated statistically to determine the applicability of this approach as a screening method for tenofovir. From the results in Figure 8, the values depicted a linear relationship between the concentration of the Au/dextran and the peak area of adenine across. The high R^2^ and low SD and RSD show that the measurements are reliable in terms of accuracy and precision. 

## 4. Materials and Methods

### 4.1. Coating Gold Nanoparticles on Glass Using the Self-Assembly Method

Sodium acetate (8 g, Sigma: S2889, St. Louis, MO, USA) was dissolved in 50 mL distilled water to make a 2 M solution. A stock solution of 2 M gold (III)chloride hydrate (Sigma: 254169, St. Louis, MO, USA) was made and diluted 1:100 to make a working solution of 0.02 M. A volume of 4 mL sodium acetate solution was pipetted into a 50 mL falcon tube, followed by 1 mL of the gold solution. The gold coating solution was gently shaken and incubated in the dark for 10 min. Distilled water (25 mL) was poured into a glass slide holder, followed by addition of the coating solution. Microscope glass slides were incubated in piranha solution 1% (*v*/*v*) for 1 h and then rinsed with distilled water. The hydrophilic slides were inserted in a beaker containing the coating solution and were incubated in the dark at an angle of approximately 45 degrees overnight. After 24 h, the glass slides were rinsed once with water to remove excess gold nanoparticles and were subsequently dried in a vacuum hood for 30 min before Raman spectroscopy experiments were carried out. A Zeiss (Jena, Germany) scanning electron microscopy (SEM) was used to analyse the gold-coated slides using an accelerating voltage of 5 kV to acquire images at 1×, while a 2 kV was used to capture images at 10 and 20× magnification.

### 4.2. Synthesis of Gold/Dextran (DEAE) Nanocomposites

Gold nanoparticles were synthesized using the chemical reduction method with dextran acting as the stabilizing agent. In short, 5 g of Dextran DEAE (Sigma) was dissolved in distilled water (100 mL) to prepare 5% (*w*/*v*) to be used as a stabilizing solution. The solution was heated until boiling, and 50 mL of hydrogen tetrachloroaurate (III) hydrate (Sigma, 291566458) stock solution (0.5 g/mL) was added thereafter. The reaction mixture was boiled for 20 min until the colour of the mixtures turned deep-violet. The reaction mixture was then cooled to room temperature. Finally, Au/dextran nanocomposites were filtered and redispersed in 10 mL distilled water and stored at 4 °C. UV-Vis spectroscopy was performed to confirm the presence of the nanocomposites by obtaining the absorbance spectra.

### 4.3. Sample Preparation and SERS Signal Acquisition

The Au/dextran nanocomposite stock solution was diluted with 5% dextran solution to make 80%, 40%, and 20% samples. Then, 100 mg of acetylsalicylic acid (Sigma, 50782) was dissolved in 1 mL of each nanoparticle sample and placed on a shaker for 1 h at 100 rpm. A 100 mg/mL tenofovir stock solution was mixed 1 to 5 with each Au/dextran NP solution to prepare a final mixture of 20% tenofovir. The mixtures were placed on a shaker for an hour prior to analysis. The Au/dextran/drug samples were allowed to stand for 2 min prior to SERS analysis. Then, 100 µL of each sample was pipetted on the gold-coated slides and placed on a BWTek Raman microscope (Plainsboro, NJ, USA) for signal acquisition using a 785 nm laser, 20× objective, 105 µm spot size, operated at 20 mW power, 100 scans and 10 s per scan. All the experiments were performed in triplicate. 

### 4.4. Spectral Processing and Data Analysis

Raman spectra were obtained from the BWTek signal acquisition software and further processed using Spectragryph v1.2.15 (Hamburg, Germany). where background subtraction, baseline correction, peak fitting and spectral comparisons were made. Microsoft Excel 2022 ( Redmond, WA, USA) was used to plot histograms and linear curves (peak area vs. concentration) for qualitative and statistical analysis respectively. Statistical data is provided in Table S1 of the Supplementary Materials.

## 5. Conclusions

The findings from this study showed how SERS can be applied in quality control of aspirin and tenofovir. Qualitative experiments provided insight on how these drugs behave in the presence of nanoparticles such as Au/dextran, which provide signal amplification via plasmonic effects. The signal enhancement support provided by the gold nanoparticle scaffolds also play a role in improving the Raman signal. Although experiments on molecular orientation would provide more correlative data, the trends that developed from SERS analysis agree with the molecular properties of both drugs in terms of intermolecular interactions that are driven by their unique combination of functional groups. Statistical results proved that the combination of nano scaffolds and Au/dextran nanoparticle solutions can detect and amplify molecular groups in a linear and precise fashion, providing a potential use in screening of substandard medication for routine quality control in a simplified and cost-effective approach. Such a method would be useful in the evaluation of aspirin and tenofovir that is supplied to third-world countries where the availability of state-of-the-art equipment and quality medication is limited.

## Figures and Tables

**Table 1:** Summary of SERS data on ASA using Au/dextran nanoparticles.

Raman Shift (cm^−1^)	Literature	Peak Assignment [33,34]
552	551	r CO_2_
707	705	δ CH
809	837	δ CH
1011	1014	δ CH, ν CO (ester)
1036	1045	Aromatic ring breathing
1146	1146	δ CH
1197	1191	δ CH
1251	1223	ν CO, acid
1297	1293	ν CO, acid
1349	1367	ν CC
1585	1576	ν CC, Aromatic

r, rocking; δ, bending; ν, stretching.

**Table 2:** Summary of SERS shifts of tenofovir using Au/dextran nanoparticles.

Raman Shift (cm^−1^)	Literature	Peak Assignment [35,36]
533	537	δ CCC
600	614	δ NCC
718	725	Adenine ring
864	841	Skeletal breathing
1011	1014	δ CH, ν CO (ester)
1014	1007	Phosphate
1014	1045	Aromatic ring breathing
1260	1255	ν CN, CNH_2_
1318	1311	ν CN
1391	1370	δ CH_3_
1557	1517	δ CNH

δ, bending; ν, stretching.

## Data Availability

Data are available via personal communication with proper reasons.

## Supplementary Materials

The following supporting information can be downloaded at: https://www.mdpi.com/article/10.3390/molecules27082554/s1, Table S1. Statistical data of Aspirin and Tenofovir.
