# Supplementary material for: A Molecular Study of Aspirin and Tenofovir Using Gold/Dextran Nanocomposites and Surface-Enhanced Raman Spectroscopy
Source: Molecules. 2022 Apr 15;27(8):2554. doi: 10.3390/molecules27082554 (PMC9029789; doi:10.3390/molecules27082554)
Supplement: Supplementary file 1 [file molecules-27-02554-s001.zip › molecules-1609723-supplementary.pdf]

molecules

MDPI

Supplementary Materials

A Molecular Study of Aspirin and Tenofovir Using Gold/Dextran Nanocomposites and Surface-Enhanced Raman Spectroscopy

Setumo Lebogang Thobakgale [1,2,*], Saturnin Ombinda-Lemboumba [1] and Patience Mthunzi-Kufa [1,2]

[1] National Laser Centre, Council for Scientific and Industrial Research, P.O. Box 395, Pretoria 0001, South Africa; sombindalemboumba@csir.co.za (S.O.-L.); pmthunzikufa@csir.co.za (P.M.-K.)
[2] School of Chemistry and Physics, College of Agriculture, Engineering and Science, University of Kwa-Zulu Natal, University Road, Westville, Durban 3630, South Africa
* Correspondence: lthobakgale@csir.co.za; Tel.: +27-12-841-3845 or +27-73-034-7403

Table S1. Statistical data of Aspirin and Tenofovir.

	**Mean (n = 3)**	**SD (±)**	**RSD (%)**
**Acetylsalicylic acid (ASA)**			
**CO (ester)**			
5% Dextran (blank)	1098	2.52	0.229
5% Dextran,10% (*w*/*v*) ASA	4109	2.08	0.051
20% (*v*/*v*) Au/Dextran,10% (*w*/*v*) ASA	8906	1.53	0.017
40% (*v*/*v*) Au/Dextran,10% (*w*/*v*) ASA	10950	1.73	0.016
80% (*v*/*v*) Au/Dextran,10% (*w*/*v*) ASA	16140	1.53	0.0101
100% (*v*/*v*) Au/Dextran,10% (*w*/*v*) ASA	16530	1.15	0.0066
**Tenofovir Disoproxil fumarate**			
**Adenine ring**			
5% Dextran (blank)	1149	3.06	0.266
5% Dextran,20% (*v*/*v*) Tenofovir	4005	1.15	0.029
20% (*v*/*v*) Au/Dextran,20% (*v*/*v*)Tenofovir	6043	2.52	0.0417
40% (*v*/*v*) Au/Dextran,20% (*v*/*v*) Tenofovir	7565	2.89	0.038
80% (*v*/*v*) Au/Dextran,20% (*v*/*v*) Tenofovir	9248	2.00	0.0216
100% (*v*/*v*) Au/Dextran,20% (*v*/*v*) Tenofovir	11710	2.65	0.0226
